# Supplementary figures and images for: Supplemental Far-Red Light Stimulates Lettuce Growth: Disentangling Morphological and Physiological Effects
Source: Plants (Basel). 2021 Jan 16;10(1):166. doi: 10.3390/plants10010166 (PMC7829796; doi:10.3390/plants10010166)

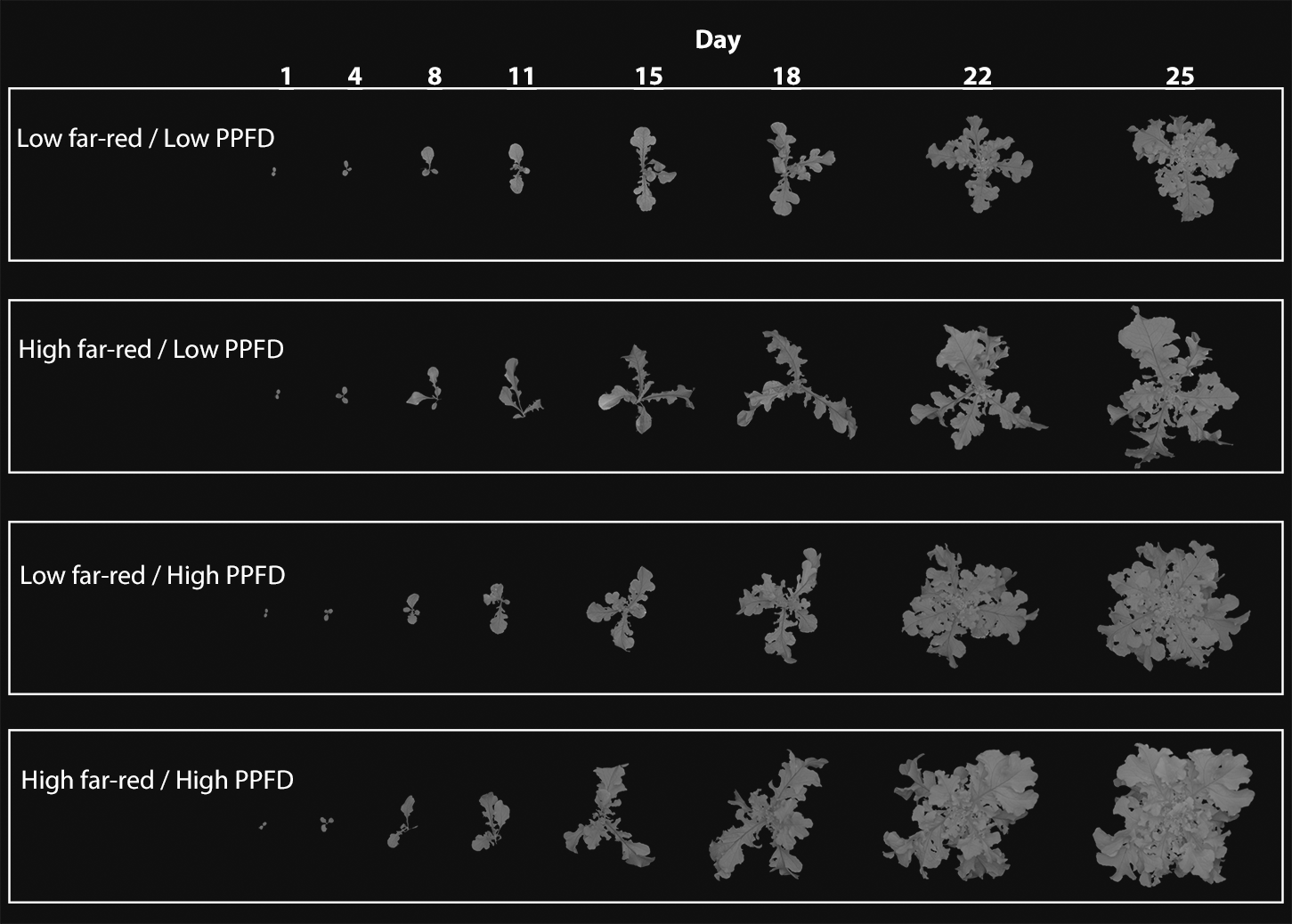

Supplement: Supplementary file 1 [file plants-10-00166-s001.zip › supplementary/SupFig1_f.png]

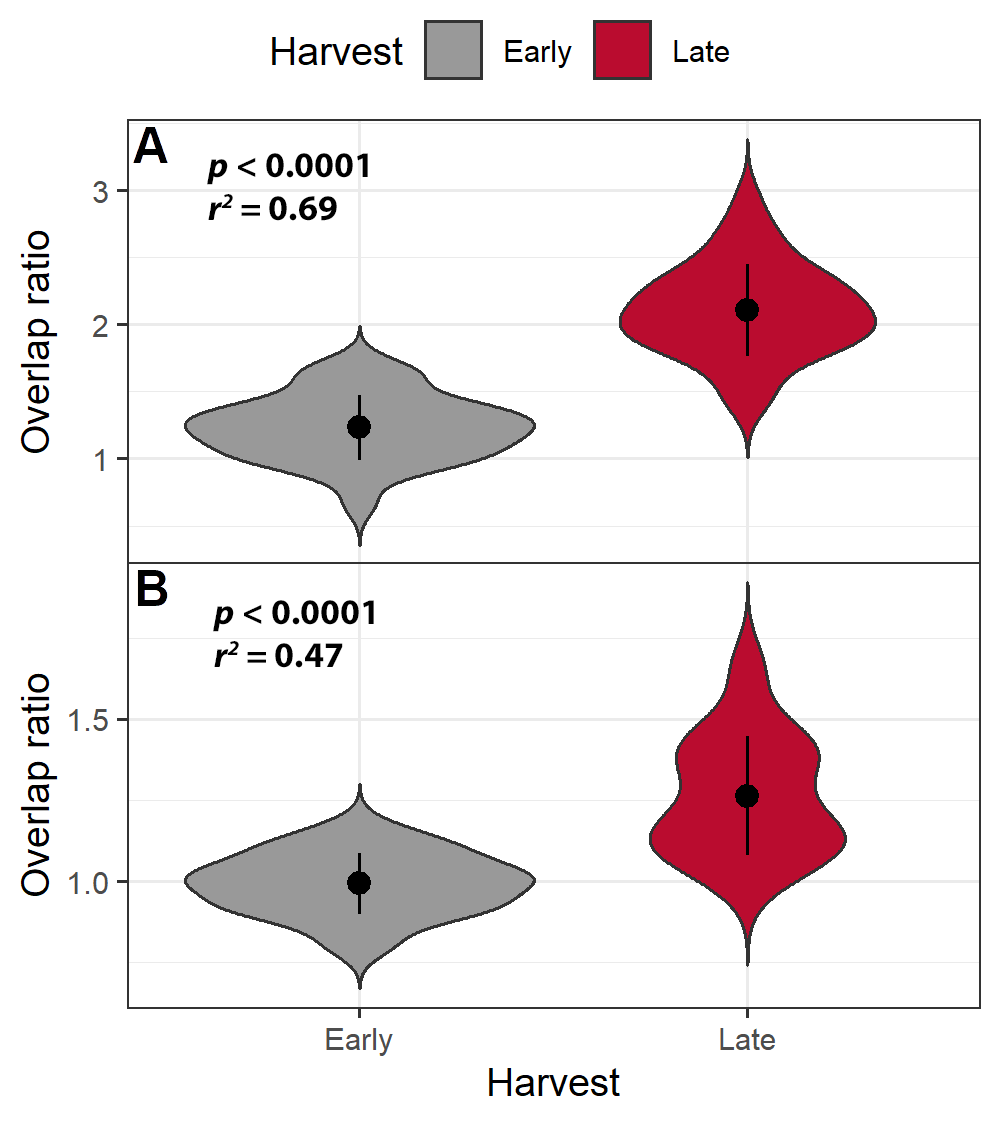

Supplement: Supplementary file 1 [file plants-10-00166-s001.zip › supplementary/SupFig2_f.png]

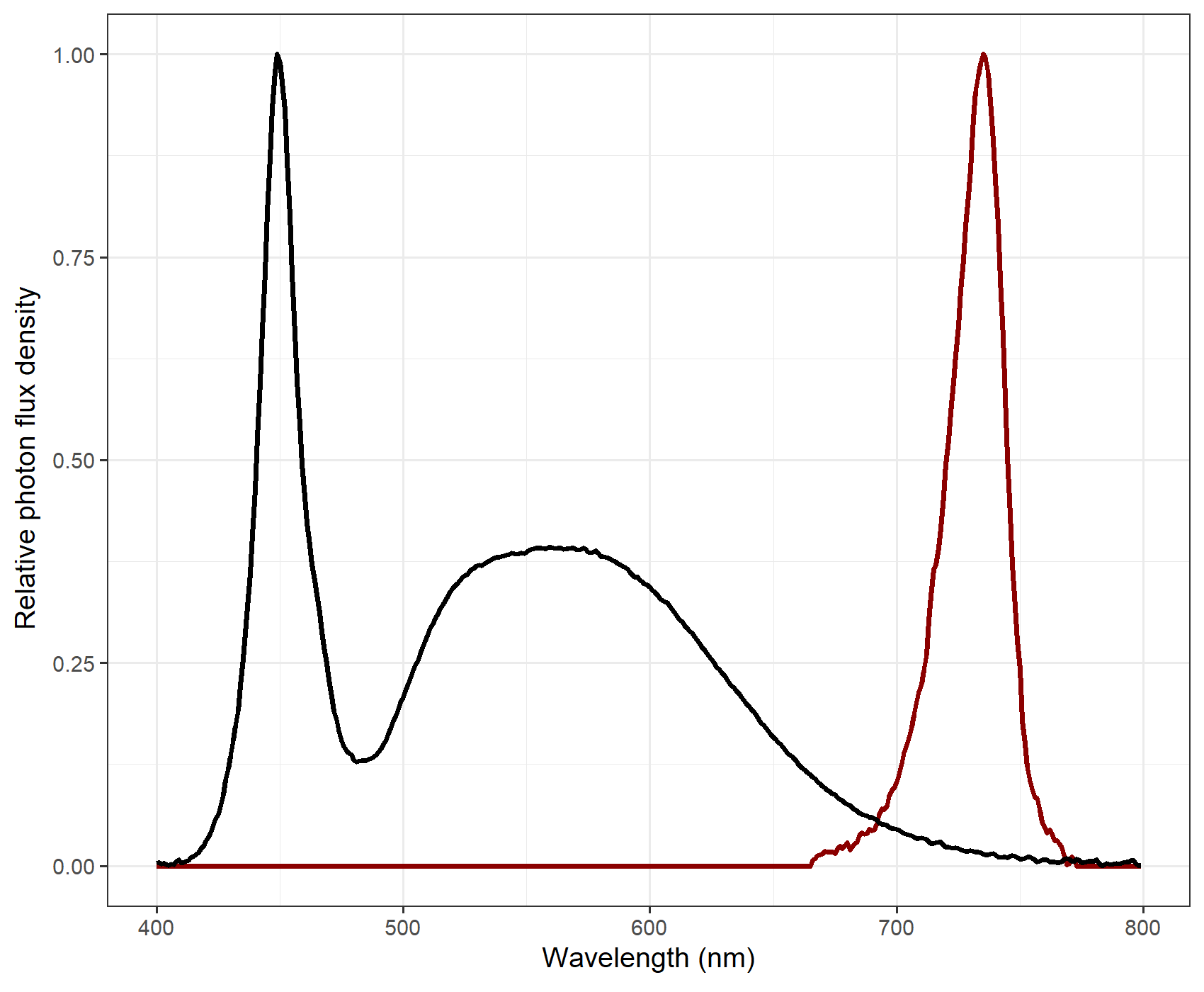

Supplement: Supplementary file 1 [file plants-10-00166-s001.zip › supplementary/SupFig3_f.png]

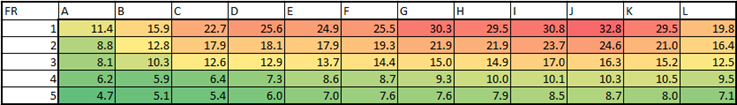

Supplement: Supplementary file 1 [file plants-10-00166-s001.zip › supplementary/SupFig4_f.png]

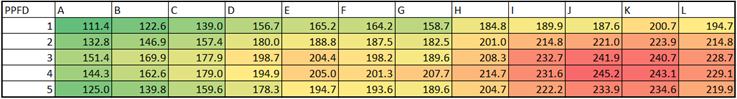

Supplement: Supplementary file 1 [file plants-10-00166-s001.zip › supplementary/SupFig5_f.png]
